# Integrated Transcriptomic and Metabolomic Analyses Reveal Dose−Dependent Effects of Bamboo Compost on Tomato Growth and Resistance to *Tuta absoluta*

**DOI:** 10.3390/ijms27188209

**Published:** 2026-09-15

**Authors:** Renbin Tu, Limin Chen, Yiming Pan, Yuhan Pan, Gul Hina, Aiwu Jin, Yaobin Lu, Farman Ullah, Qian Xu, Yikun Wang, Zhe Chen, Xianjun Deng, Liangjian Hu, Xiaowei Li, Qianggen Zhu

**Affiliations:** 1 College of Agriculture and Biotechnology, Lishui University, Lishui 323000, China; turenbin0026@163.com (R.T.); clmit@zju.edu.cn (L.C.); kinaw@zafu.edu.cn (A.J.); xuqian32@126.com (Q.X.); wykknjfu@163.com (Y.W.); czhe1108@foxmail.com (Z.C.); xianjundeng@126.com (X.D.); xiaoka_777@foxmail.com (L.H.); 2 State Key Laboratory for Quality and Safety of Agro-Products, Key Laboratory of Biotechnology in Plant Protection of the Ministry of Agriculture and Rural Affairs, Institute of Plant Protection and Microbiology, Zhejiang Academy of Agricultural Sciences, Hangzhou 310021, China; pym9248@163.com (Y.P.); panyuh0306@163.com (Y.P.); gulhina680@gmail.com (G.H.); luybcn@163.com (Y.L.); farmanullah787@gmail.com (F.U.); 3 Lishui Institute of Agriculture and Forestry Sciences, Lishui 323000, China; 4 State Key Laboratory of Agricultural and Forestry Biosecurity, Ministerial and Provincial Joint Innovation Centre for Safety Production of Cross-Strait Crops, Fujian Agriculture and Forestry University, Fuzhou 350002, China; 5 China National Bamboo Research Center, Hangzhou 310012, China

**Keywords:** bamboo compost, tomato, *Tuta absoluta*, transcriptomics, metabolomics, peat substitution, growth–defense trade-off

## Abstract

Bamboo compost is a renewable organic material that can replace peat and enhance plant resistance to insect pests. However, its combined effects on tomato (*Solanum lycopersicum* *L*.) growth, defense responses, and underlying molecular mechanisms remain unclear. In this study, bamboo compost was incorporated into growing substrates at varying proportions (5–30%) to assess its impact on tomato seedling growth, physiological and biochemical traits, and the survival of the South American tomato leafminer, *Tuta absoluta*. Metabolomic and transcriptomic analyses were further integrated to characterize molecular changes associated with enhanced resistance. The results showed that 10% bamboo compost increased plant height and stem diameter by 25.91% and 10.19%, respectively, while higher proportions inhibited seedling growth. All bamboo compost treatments significantly reduced *Tuta absoluta*. larval survival, with the lowest day-10 survival (16%) observed under BC−20%. Multi-omics analyses identified 1553 and 2591 DEGs and 292 and 591 DAMs in the BC−10% vs. CK and BC−20% vs. CK comparisons, respectively; 290 differentially expressed transcription factor-encoding genes were identified across both comparisons. BC−20% showed broader defense-related transcriptional and metabolic reprogramming, including MAPK and phytohormone signaling, phenylpropanoid and flavonoid biosynthesis, and cutin and wax formation, but with reduced growth. Overall, BC−10% represents a suitable practical addition rate for promoting tomato seedling growth in peat-reduced cultivation substrates.

## 1. Introduction

Tomato (*Solanum lycopersicum* *L*.) is one of the world’s most economically important vegetable crops and is widely consumed both fresh and as processed products. Beyond its agricultural value, tomato fruits are important sources of vitamins, carotenoids, particularly lycopene, and phenolic compounds that contribute to their nutritional quality and potential health-promoting properties. Therefore, sustainable tomato production and crop protection are relevant to both agricultural productivity and public health [1,2]. Modern agriculture and forestry produce large amounts of lignocellulosic organic solid waste each year. Improper disposal of plant-derived residues−such as crop straw, crop residues, and landscaping waste−results in resource loss, greenhouse gas emissions, and environmental pollution [3,4]. Thus, valorization, volume reduction, and low-carbon recycling of these wastes have become major challenges for sustainable agriculture and the circular economy [5,6].

Conventional horticultural nurseries and greenhouse vegetable production have long depended on peat-based growing media. However, peat is a slowly renewable wetland carbon resource that forms over thousands of years. Its extraction and use contribute to carbon emissions, peatland degradation, and concerns about resource sustainability [7,8]. Consequently, developing environmentally friendly growing media from renewable biomass—including agricultural and forestry residues, landscaping waste, wood fibers, composts, and agricultural by-products—has become a key strategy for enhancing the sustainability of protected horticulture [9,10].

Bamboo resources are abundant in China and other parts of Asia, and bamboo processing produces large amounts of chips, powder, and other residues. Owing to their high lignocellulose content and structural stability, bamboo residues have significant potential for conversion into materials, carbon-based products, and horticultural growing media components [11,12]. Nevertheless, untreated bamboo residues and other lignocellulosic wastes are generally highly lignified, decompose slowly, and have imbalanced C/N ratios. Their direct application may therefore cause nitrogen immobilization, phytotoxicity, or root stress [13,14]. Efficient aerobic composting, microbially enhanced composting, and appropriate pretreatment can mitigate phytotoxicity and promote organic-matter stabilization and mineral-nutrient release [15,16]. Composting also improves substrate porosity, water-holding capacity, and aeration, creating a more favorable rhizosphere environment [17,18]. Studies have shown that woody compost, agricultural waste compost, biochar−compost mixtures, and wood fiber−based media can partially replace peat. These alternatives improve seedling growth, root development, nutrient uptake, and yields in crops such as tomato, cucumber, strawberry, and lettuce [19,20].

Of particular interest is the potential of organic-waste-derived growing materials to act as “functional growing media” that both support plant growth and induce defense responses. Organic amendments may enhance plant resistance to pathogens, nematodes, and herbivorous insects by modulating rhizosphere microbial communities, releasing humic-like signaling compounds, improving nutritional status, and activating immune−related pathways [21,22]. Humic biostimulants, compost teas, and microbial inoculants can alter primary and secondary metabolism, improving stress tolerance and overall plant health [23,24]. Likewise, organic amendments such as vermicompost and biochar have been reported to enhance plant growth, soil biological activity, and tolerance to biotic stress [25,26]. For instance, combined application of biochar, compost, and plant-growth-promoting bacteria suppressed early blight and improved tomato growth. Reviews on the suppressive effects of biochar on above and belowground pests and diseases further suggest that these benefits are closely associated with altered substrate physicochemical properties, microbial community restructuring, and induced plant resistance [27,28].

Evidence for the role of organic growing media in insect pest management is also accumulating. Greater organic-matter inputs or altered substrate conditions can exert bottom–up effects on plant nutrition, defensive metabolism, and herbivore performance [29,30]. Incorporation of bamboo charcoal into growing media has been shown to promote tomato growth and reduce survival of the South American tomato leafminer (*Tuta absoluta*) by increasing defensive metabolites such as flavonoids, terpenoids, and phenolic acids [31]. Another study on bamboo charcoal and the whitefly *Bemisia tabaci* found that bamboo-charcoal treatment increased tomato plant height, stem diameter, and concentrations of free amino acids, starch, and soluble sugars while reducing whitefly survival, oviposition, and field population density [32]. Unlike bamboo charcoal, which is produced by pyrolysis and is relatively carbon-stable, bamboo compost is generated through aerobic microbial decomposition. It may therefore differ in labile nutrient supply, soluble organic constituents, and rhizosphere-associated processes. Comparative studies have shown distinct nutrient-release patterns and microbial responses between compost and biochar [33,34]. In bamboo systems, composted bamboo residues can alter soil microbial−community composition and can serve as a partial peat substitute in horticultural substrates [35,36]. Moreover, exogenous phenylalanine enhanced tomato resistance to *Tuta absoluta* by promoting accumulation of phenylpropanoid- and benzoic-acid-derived volatiles. Metabolomic analyses of wild tomato resistance to *B. tabaci* and *Tuta absoluta* have also highlighted the importance of fatty acids and related metabolic pathways in insect resistance [37,38]. Collectively, these findings indicate that organic growing media derived from agricultural waste may serve not only as physical substrates and nutrient sources but also as regulators of plant quality, defensive metabolism, and volatile emissions, thereby affecting insect feeding, survival, and reproduction [39,40].

Here, we evaluated the dose-dependent effects of bamboo compost incorporated into a peat-reduced tomato substrate on plant growth, physiological and biochemical traits, *Tuta absoluta* survival, and leaf transcriptomic and metabolomic profiles. Building on previous bamboo-charcoal studies, our aim was not to establish a compost-exclusive defense mechanism, but to determine whether bamboo compost produces a dose-dependent growth–defense pattern in a peat-substrate context. These omics approaches, together with volatilomic profiling, have become valuable tools for deciphering defense−regulatory networks in tomato and other plant species exposed to pathogen infection, insect herbivory, and environmental stress [41]. Comparable integrated analyses in rice and cucumber have shown that herbivory coordinates phytohormone signaling with primary and secondary metabolism, including phenylpropanoid and terpenoid pathways [42,43]. These cross-crop findings support the use of transcriptome−metabolome integration to investigate bamboo-compost-associated growth–defense responses in tomato. Growth and induced defense may involve resource allocation trade-offs, in which stronger investment in defense-related metabolism can be associated with constrained biomass accumulation [44]. Accordingly, we hypothesized that bamboo compost would produce a dose-dependent growth−defense response: BC−10% would favor tomato seedling growth, whereas higher proportions would be associated with reduced growth but stronger defense-related reprogramming. We further predicted dose-dependent responses in MAPK signaling, jasmonate-associated phytohormone signaling, and phenylpropanoid/flavonoid metabolism. Thus, the principal novelty of this study lies in defining the dose-dependent growth–defense response associated with bamboo compost in a peat-reduced tomato substrate. This study provides a theoretical and practical basis for the valorization of bamboo-derived organic waste, the development of peat-reduced growing media, and the sustainable management of *Tuta absoluta*.

## 2. Results

### 2.1. Effects of Different Proportions of Bamboo Compost in Growing Substrates on Tomato Growth

**Figure 1:**
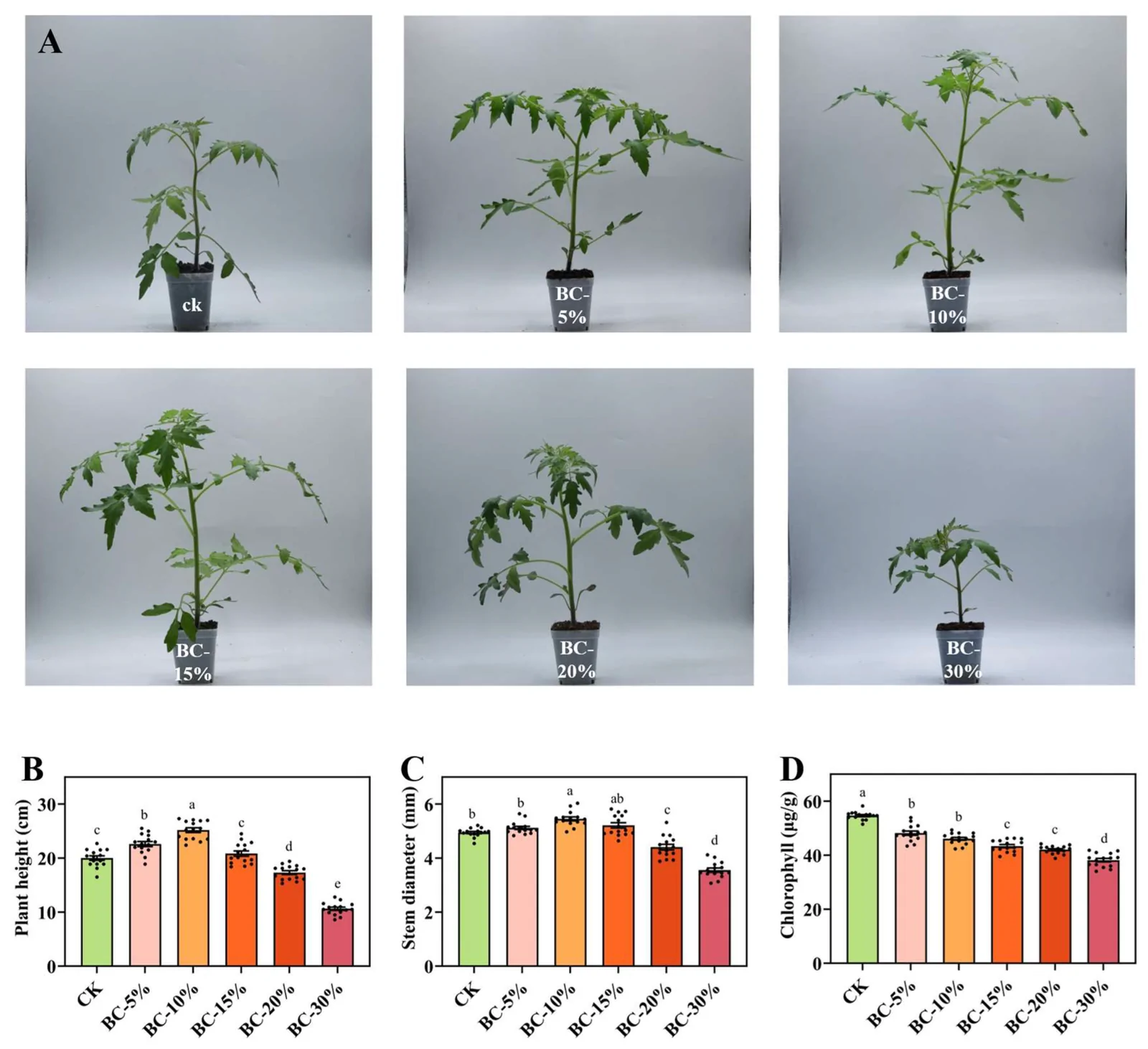
Effects of different bamboo compost proportions in cultivation substrates on tomato growth. (**A**) Growth status of potted tomato plants under different treatments on 11 April 2026. (**B**) Comparison of potted tomato plant height among different treatments on 11 April 2026 (n = 15 plants, Date 11 April 2026: *F*_5,84_ = 149.918, *p* < 0.001). (**C**) Comparison of potted tomato stem diameter among different treatments on 11 April 2026 (n = 15 plants, Date 11 April 2026: *F*_5,84_ = 92.599, *p* < 0.001). (**D**) Comparison of chlorophyll content of potted tomato among different treatments on 11 April 2026 (n = 15 plants, Date 11 April 2026: *F*_5,84_ = 99.796, *p* < 0.001). Differences among means (±SE) were analyzed using one-way ANOVA followed by Tukey’s HSD test analysis. Different letters indicate significant differences among treatments (*p* < 0.05).

At 20 days after transplanting, the plant height of tomato seedlings increased by 13.10%, 25.91%, and 4.20% in the BC−5%, BC−10%, and BC−15% treatments, respectively, compared to the control (CK). In contrast, the BC−20% and BC−30% treatments resulted in significant decreases of 13.40% and 46.85%, respectively (Figure 1B, Supplementary Table S1a). A similar pattern was seen in stem diameter: the BC−5%, BC-10%, and BC−15% treatments increased stem diameter by 3.44%, 10.19%, and 5.32%, respectively, while the BC−20% and BC−30% treatments reduced it by 10.92% and 28.14% (Figure 1C, Supplementary Table S1a). These results indicate that the BC−10% treatment was most beneficial for tomato seedling growth. However, chlorophyll content in all bamboo compost treatments was significantly lower than in the CK group at 20 days after transplanting, with the degree of reduction varying among treatments (Figure 1D, Supplementary Table S1a). The lower chlorophyll concentration under BC−10%, despite improved plant height and stem diameter, may reflect dilution associated with enhanced leaf expansion, whereas reductions at higher incorporation rates may be related to substrate-mediated changes in nutrient availability. These possibilities require further verification because whole-plant chlorophyll and leaf biomass were not determined. Overall, considering plant height, stem diameter, and chlorophyll content, BC−10% was identified as the optimal substrate for promoting tomato seedling growth (Figure 1A).

### 2.2. Survival of Tuta absoluta on Leaves of Tomato Plants Grown in Bamboo-Compost-Amended Substrates

**Figure 2:**
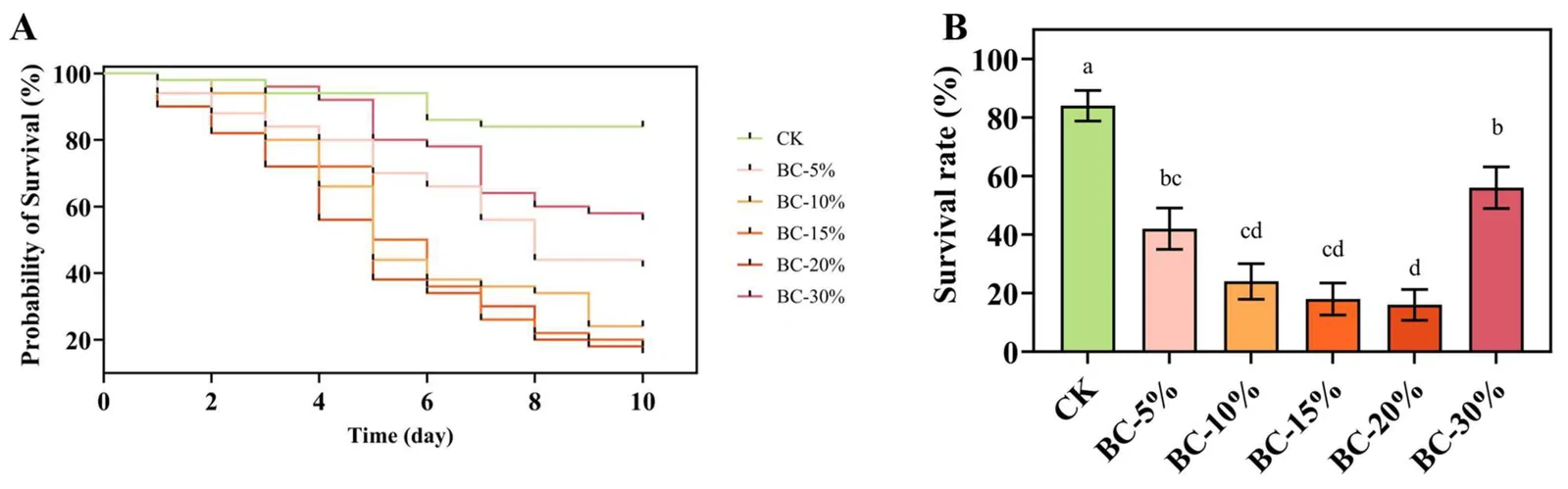
Effects of different ratios of bamboo compost added to the growing substrate on tomato resistance to pests. (**A**) Survival curves of *Tuta absoluta* on tomato leaves from substrates with different bamboo compost ratios (n = 300, log-rank test: *χ*^2^ = 73.60, *df* = 5, *p <* 0.0001). (**B**) Survival rates of *Tuta absoluta* on tomato plants grown in substrates with different bamboo compost ratios (n = 5, *F*_5,294_ = 18.9664, *p* < 0.0001). Survival curves in (**A**) were compared using the log-rank test. Day-10 survival rates in (**B**) were analyzed using one-way ANOVA followed by Tukey’s HSD test. Different letters indicate significant differences among treatments (*p* < 0.05).

The survival curves of *Tuta absoluta* larvae differed significantly among the bamboo compost treatments (log rank test: *χ*^2^ = 73.60, df = 5, *p* < 0.0001; Figure 2A, Supplementary Table S1b). At day 10, the survival rate of larvae reared on leaves from tomato plants treated with bamboo compost was significantly lower than that of larvae reared on leaves from control (CK) plants (*F*_5,294_ = 18.966, *p* < 0.0001). At day 10, larval survival was 84%, 42%, 24%, 18%, 16%, and 56% in the CK, BC−5%, BC−10%, BC−15%, BC−20%, and BC−30% treatments, respectively (Figure 2B; Supplementary Table S1b). Collectively, these results indicate that bamboo compost treatment was associated with enhanced resistance to *Tuta absoluta*, as reflected by reduced larval survival.

### 2.3. Effects of Different Bamboo Compost Proportions on Biochemical Parameters in Tomato Plants

**Figure 3:**
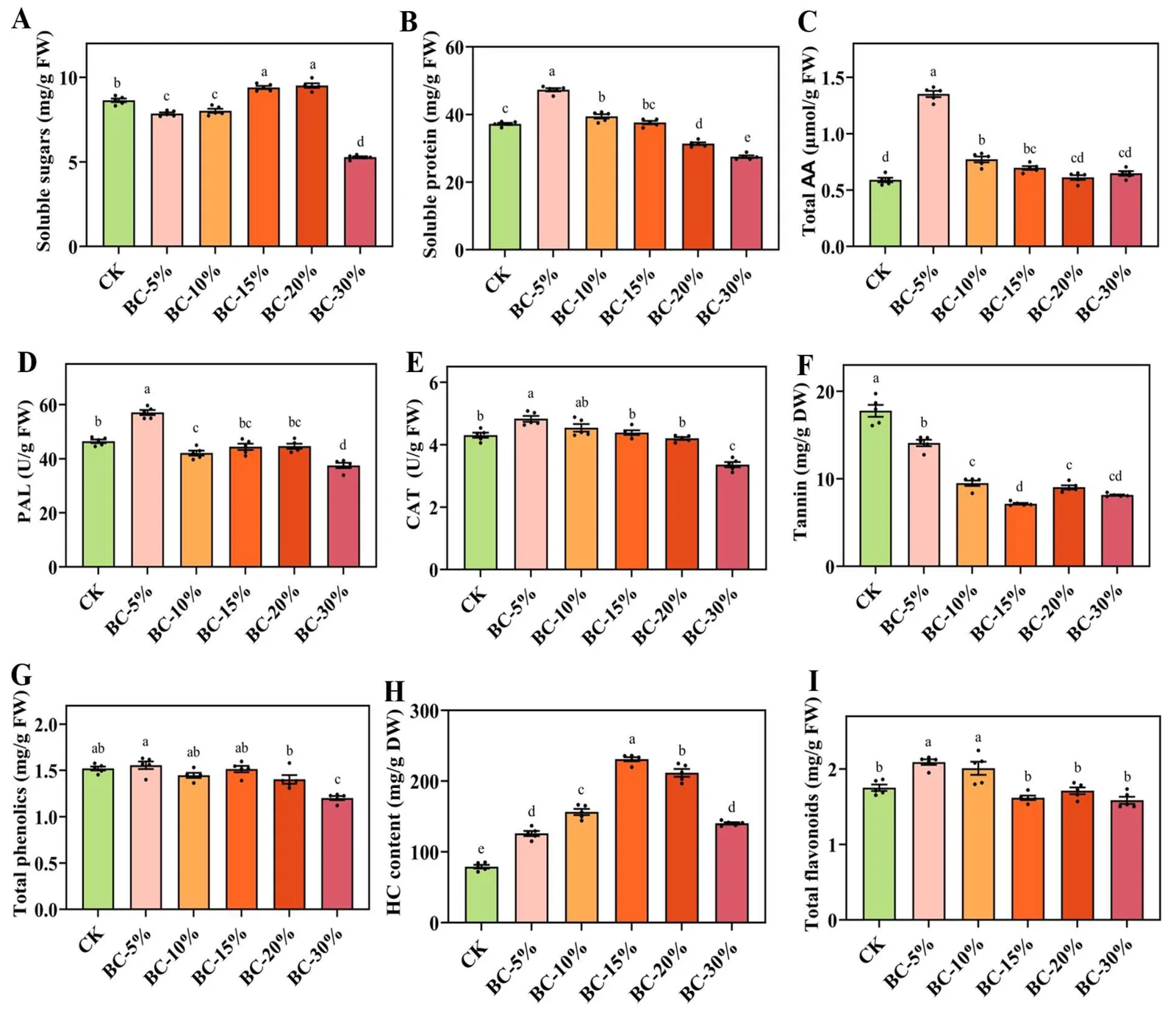
Effects of different proportions of bamboo compost added to tomato cultivation substrate on the physiological and biochemical parameters of tomato. (**A**) Soluble sugar (n = 5, *F*_5,24_ = 236.335, *p* < 0.001). (**B**) Soluble protein (n = 5, *F*_5,24_ = 213.445, *p* < 0.001). (**C**) Total amino acid (n = 5, *F*_5,24_ = 171.332, *p* < 0.001). (**D**) PAL activity (n = 5, *F*_5,24_ = 47.203, *p* < 0.001). (**E**) CAT activity (n = 5, *F*_5,24_ = 32.930, *p* < 0.001). (**F**) Tannin content (n = 5, *F*_5,24_ = 131.935, *p* < 0.001). (**G**) Total phenolics (n = 5, *F*_5,24_ = 15.085, *p* < 0.001). (**H**) Hemicellulose (n = 5, *F*_5,24_ = 241.442, *p* < 0.001). (**I**) Total flavonoids (n = 5, *F*_5,24_ = 16.476, *p* < 0.001). Different lowercase letters indicate significant differences among treatments (one-way ANOVA, Tukey’s HSD test, *p* < 0.05).

Biochemical parameters of tomato plants varied significantly among treatments (Figure 3). Compared with the control (CK), bamboo-compost (BC) treatments significantly increased the contents of soluble sugars (Figure 3A, Supplementary Table S2), soluble proteins (Figure 3B, Supplementary Table S2), and total amino acids (Figure 3C, Supplementary Table S2) (soluble sugars: *F*_5,24_ = 236.335, *p* < 0.001; soluble proteins: *F*_5,24_ = 213.445, *p* < 0.001; total amino acids: *F*_5,24_ = 171.332, *p* < 0.001). Soluble protein and total amino acid contents peaked in the BC−5% treatment, whereas soluble sugar contents were relatively higher in the BC−15% and BC−20% treatments.

Bamboo compost also had a significant impact on phenylalanine ammonia-lyase (PAL) and catalase (CAT) activities (PAL: *F*_5,24_ = 47.203, *p* < 0.001; CAT: *F*_5,24_ = 32.930, *p* < 0.001; Figure 3D,E, Supplementary Table S2). Both enzyme activities reached their highest levels in the BC−5% treatment and generally decreased as the proportion of bamboo compost increased. Compared to the control (CK), PAL activity was significantly reduced in the BC−10% and BC−30% treatments, while CAT activity was decreased only in the BC−30% treatment.

Tannin content was significantly lower in all BC treatments than in CK (*F*_5,24_ = 131.935, *p* < 0.001; Figure 3F, Supplementary Table S2). The effects of BC treatments on other phenolic compounds varied. Total flavonoid content was significantly increased by the BC−5% and BC−10% treatments (*F*_5,24_ = 16.476, *p* < 0.001; Figure 3I, Supplementary Table S2), whereas total phenolic content did not differ significantly from CK in most treatments and was reduced only in the BC−30% treatment (Figure 3G, Supplementary Table S2). Notably, all BC treatments significantly increased hemicellulose content (*F*_5,24_ = 241.442, *p* < 0.001; Figure 3H, Supplementary Table S2), which exhibited a clear upward trend with increasing bamboo-compost proportions.

### 2.4. Metabolomic Responses of Tomato Plants Grown in Bamboo-Compost-Amended Substrates

**Figure 4:**
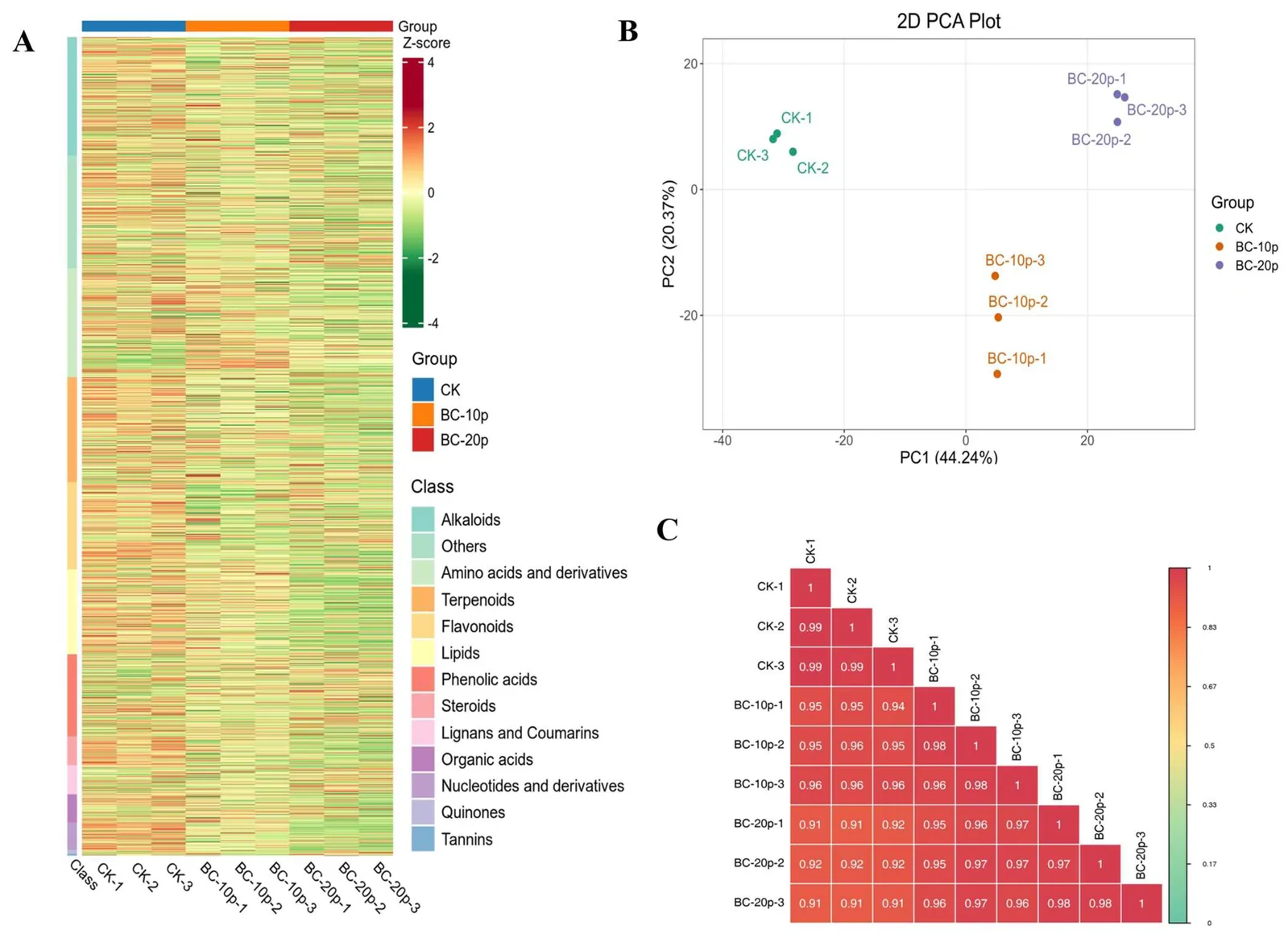
Metabolomic analysis of tomato leaves in BC−10%, BC−20% and CK groups. (**A**) Heatmap of the relative intensities of detected metabolites across different treatment groups. (**B**) Principal component analysis. (**C**) Pearson’s correlation coefficients of metabolite relative intensities among all treatment groups.

In total, 2861 metabolites were detected in the metabolomic profiles of tomato leaves across treatment groups. These metabolites were classified into alkaloids, amino acids and their derivatives, terpenoids, flavonoids, lipids, phenolic acids, steroids, lignans and coumarins, organic acids, nucleotides and their derivatives, quinones, and tannins (Figure 4A). Principal component analysis (PCA) revealed tight clustering of biological replicates within the CK, BC−10%, and BC−-20% groups (Figure 4B). Furthermore, all Pearson’s correlation coefficients (PCCs) exceeded 0.91 (Figure 4C). Collectively, the PCA and correlation analyses confirmed the high reproducibility and reliability of the metabolomic dataset.

### 2.5. Differential Metabolite Profiles of Tomato Leaves Between the Control and Bamboo Compost Treatments

**Figure 5:**
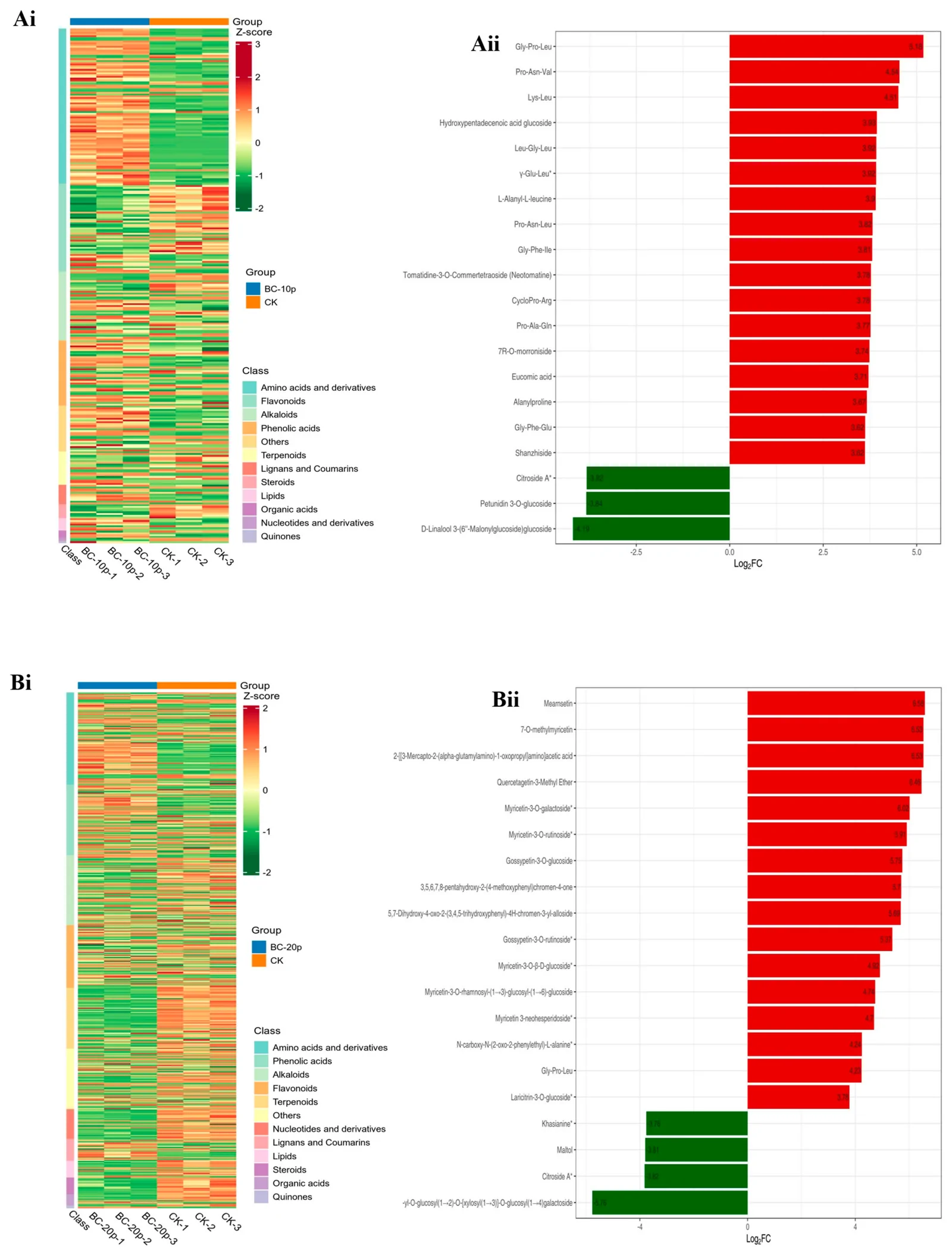
Differential metabolomic profiles across CK, BC-10% (10% bamboo compost supplementation) and BC−20% (20% bamboo compost supplementation) treatment groups. (**Ai**) Clustering heatmap of differential metabolites between BC−10% and CK. (**Aii**) Top 10 up- and down-accumulated metabolites in the BC−10% vs. CK comparison. (**Bi**) Clustering heatmap of differential metabolites between BC−20% and CK. (**Bii**) Top 10 up- and down-accumulated metabolites in the BC−20% vs. CK comparison. The asterisk (*) indicates that the compound has isomers. Since mass spectrometry cannot distinguish between isomers, the asterisk (*) is used to denote this.

Using a variable importance in projection (VIP) score > 1 and an absolute log_2_ fold change ≥ 1.0 as screening criteria, we identified a total of 292 differentially accumulated metabolites (DAMs) between the BC−10% and CK groups (Supplementary Table S3a). These DAMs were classified into amino acids and their derivatives, flavonoids, alkaloids, phenolic acids, terpenoids, lignans and coumarins, steroids, lipids, organic acids, nucleotides and their derivatives, quinones, and other compounds (Figure 5Ai). Among them, 44 metabolites were detected exclusively in BC−10% leaves relative to CK, predominantly comprising amino acids and their derivatives (18), terpenoids (6), alkaloids (4), and flavonoids (3). In addition to these 44 metabolites, 10 compounds—including eight amino acids and their derivatives, one phenolic acid, and one terpenoid—showed significantly higher accumulation in BC−10% than in CK (Figure 5Aii). Most of the remaining metabolites with elevated levels in BC−10% were amino acids and their derivatives, phenolic acids, alkaloids, and flavonoids. These results indicate that cultivation in a substrate containing 10% bamboo compost was associated with higher abundances of these metabolite classes in tomato leaves.

Applying the same criteria, 591 DAMs were identified between the BC−20% and CK groups (Supplementary Table S3b) and were categorized into the same compound classes (Figure 5Bi). Of these, 36 metabolites were unique to BC−20% leaves relative to CK, mainly including amino acids and their derivatives (16), terpenoids (4), flavonoids (4), alkaloids (3), and lipids (2). An additional 10 compounds—three amino acids and their derivatives and seven flavonoids—accumulated at significantly higher levels in BC−20% than in CK (Figure 5Bii). Most other metabolites showing higher abundance in BC−20% belonged to amino acids and their derivatives, phenolic acids, alkaloids, and flavonoids. These findings suggest that a substrate with 20% bamboo compost was associated with higher abundances of these metabolite classes in tomato leaves.

### 2.6. RNA Quality Assessment and Sequencing Data Statistics

To characterize molecular changes associated with bamboo compost treatment and reduced larval survival in tomato plants, the BC−10% and BC−20% treatments were selected for transcriptome sequencing based on their integrated effects on plant growth and development, biochemical composition, insect resistance, and defense-related metabolite levels. RNA sequencing was performed on samples from the control (CK), BC−10%, and BC−20% groups, with three biological replicates per treatment, totaling nine samples. After data filtering and quality control, clean reads per sample ranged from 47,268,852 to 56,943,598 in the CK group, from 46,264,384 to 53,907,356 in the BC−10% group, and from 48,894,958 to 58,162,916 in the BC−20% group. Transcriptome sequencing generated approximately 70.52 Gb of clean bases in total, with each sample yielding no less than 6.99 Gb. The percentage of bases with Q30 scores was ≥ 96.31%, and the GC content ranged from 42.63% to 42.93%. The overall mapping rate to the reference genome ranged from 99.11% to 99.28% (Table 1). These quality metrics confirmed the high quality of the sequencing data and its suitability for downstream analyses, including differential gene expression analysis. The datasets of the transcriptomes from our study were uploaded to the NCBI Sequence Read Archive with accession number PRJNA1498230.

### 2.7. Transcriptome Sequencing Analysis

**Figure 6:**
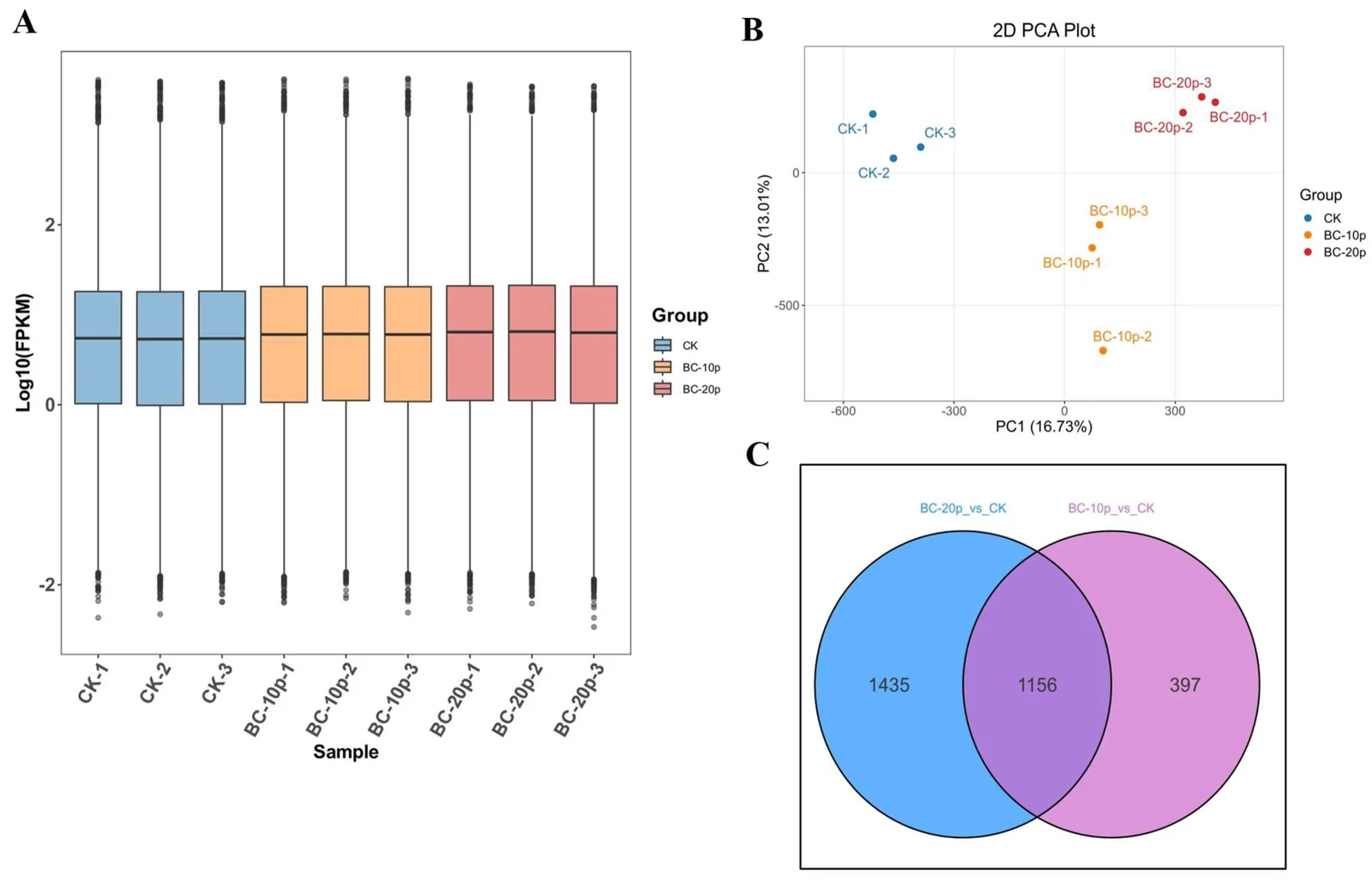
Transcriptome analysis of tomato leaves cultivated with bamboo compost (BC) at different addition ratios. (**A**) Violin plots of gene expression levels in different treatment groups. (**B**) Principal component analysis (PCA) of transcriptomic profiles from control (CK) and BC−treated samples. (**C**) Numbers of differentially expressed genes (DEGs) in each BC treatment group.

To investigate the effects of bamboo-compost incorporation on gene expression in tomato plants, we first examined the distribution of fragments per kilobase of transcript per million mapped reads (FPKM) values across samples. All biological replicates exhibited highly consistent overall gene expression patterns (Figure 6A), indicating good experimental reproducibility. Principal component analysis (PCA) was then performed to evaluate transcriptomic relationships and variation among treatment groups. The first two principal components accounted for 16.73% (PC1) and 13.01% (PC2) of the total variance, respectively (Figure 6B). The PCA score plot revealed tight clustering of biological replicates within each group and clear separation among the CK, BC−10%, and BC−20% groups, suggesting that both bamboo-compost treatments were associated with systematic transcriptomic reprogramming in tomato leaves.

Differential expression analysis identified 1553 differentially expressed genes (DEGs) in the BC−10% treatment relative to CK, of which 680 were upregulated (Supplementary Table S4). In the BC−20% treatment, 2591 DEGs were detected, with 978 upregulated (Supplementary Table S4). A total of 1156 DEGs were shared between the two treatments (Figure 6C). To further characterize the biological processes and metabolic pathways regulated by bamboo−compost amendment, we performed Gene Ontology (GO) functional annotation and Kyoto Encyclopedia of Genes and Genomes (KEGG) pathway enrichment analyses on the identified DEGs.

#### RT-qPCR Validation of DEGs Associated with Tomato Resistance to *Tuta absoluta*

**Figure 7:**
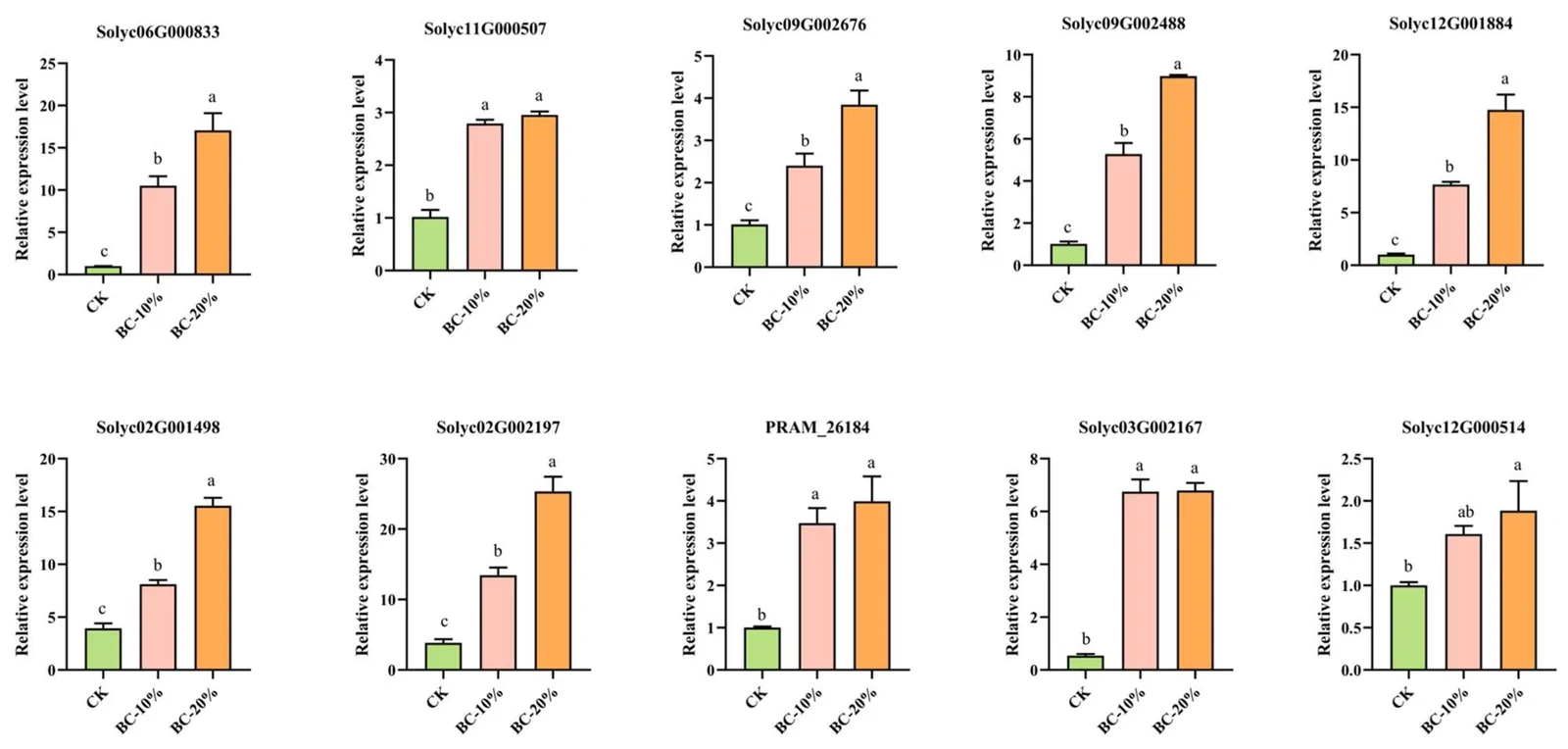
The bar chart shows the mean ± SE of the 3 biological replicates. Different lowercase letters represent significant differences at *p* < 0.05 level (one-way analysis of variance (ANOVA) with Tukey’s post hoc test).

To validate the RNA−seq results, RT−qPCR was performed for 10 selected DEGs associated with defense signaling, phenylpropanoid/flavonoid metabolism, and cuticular wax/terpenoid biosynthesis. These genes included a wound-induced proteinase inhibitor, 13S−lipoxygenase, phospholipase A1, an *LRR receptor*−*like kinas*e, *WRKY40-like*, *CHS1B*, *F3H*, *CCR*−*like*, *KCS6*, and *GGPS1*. Their expression patterns across the CK, BC−10%, and BC−20% treatments were consistent with the RNA−seq data (Figure 7), supporting the reliability of the transcriptomic analysis.

### 2.8. GO Enrichment Analysis

**Figure 8:**
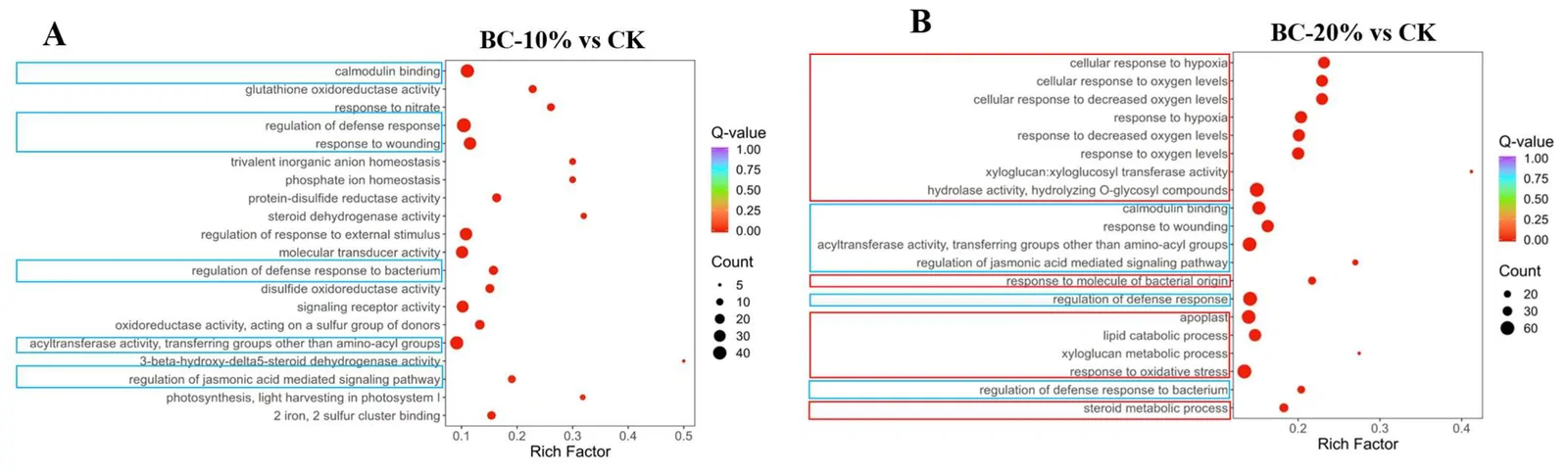
GO enrichment analysis of differentially expressed genes in tomato plants cultivated with different addition ratios of bamboo compost. (**A**) BC−10% vs. CK. (**B**) BC−20% vs. CK. Blue boxes indicate pathways commonly enriched in both BC−10% vs. CK and BC−20% vs. CK, while red boxes indicate pathways enriched in BC−20% vs. CK but not in BC−10% vs. CK. The pathways marked with boxes represent specific pathways discussed in the text, rather than the entire set of enriched GO terms.

Gene Ontology (GO) enrichment analysis was performed on the differentially expressed genes (DEGs) identified in the BC−10% and BC−20% treatments relative to the control (CK) (Supplementary Table S5). In both bamboo−compost treatments, multiple biological process (BP) and molecular function (MF) categories associated with defense response regulation, jasmonic acid signaling, and calmodulin binding were significantly enriched. These categories included regulation of defense response, response to wounding, regulation of jasmonic acid-mediated signaling pathway, and regulation of defense response to bacteria (Figure 8, blue, Supplementary Table S8). Such categories are commonly linked to early calcium-dependent signaling cascades, jasmonic acid-mediated defense pathways, and the biosynthesis of lipid− or steroid−derived defense compounds. Collectively, these findings indicate that bamboo−compost treatment was associated with transcriptional changes related to substrate-responsive and broad-spectrum plant defense.

Compared with the BC−10% treatment, the BC−20% treatment exhibited unique or stronger enrichment of GO terms associated with hypoxia−related responses, including cellular response to hypoxia and response to hypoxia, as well as lipid− and steroid−related metabolic processes, such as lipid catabolic process and steroid metabolic process (Figure 8, red, Supplementary Table S8). The enrichment of these categories suggests that the higher bamboo-compost proportion induced a broader transcriptional response, likely reflecting enhanced metabolic adaptation, respiratory burst activity, and redox regulation rather than direct hypoxic stress per se. Overall, these results indicate that increasing the bamboo−compost proportion triggered more extensive transcriptional reprogramming in tomato plants.

### 2.9. KEGG Pathway Enrichment Analysis

**Figure 9:**
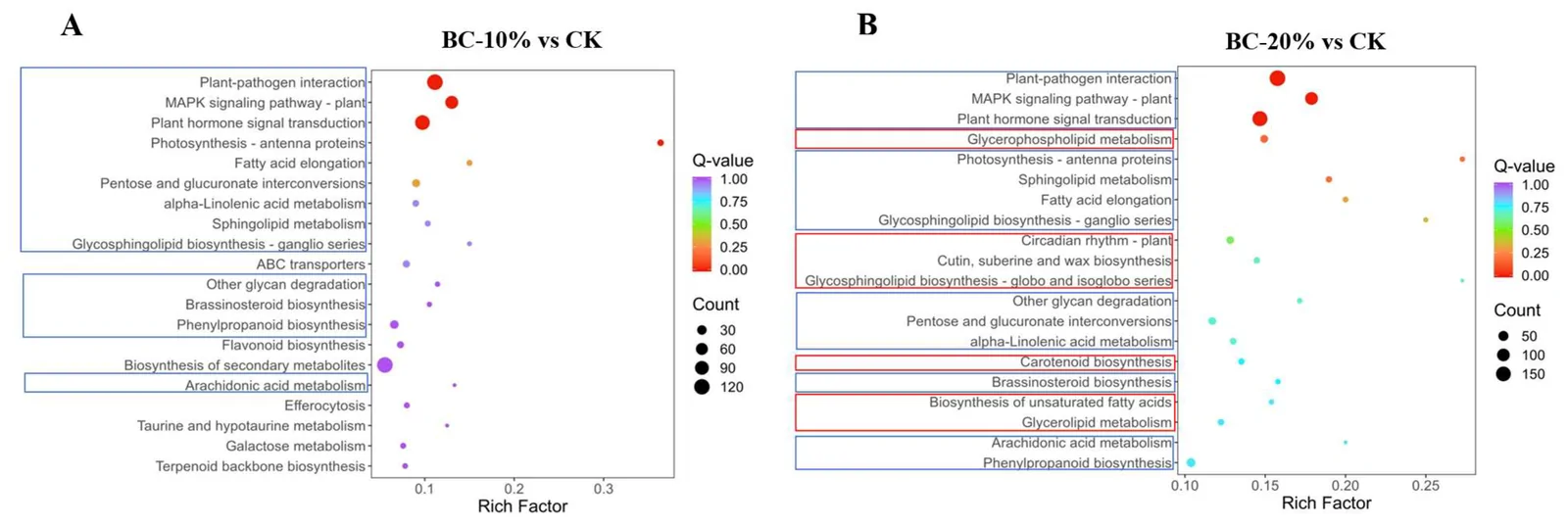
KEGG enrichment analysis of differentially expressed genes (DEGs) in tomato plants cultivated with bamboo compost at different application ratios. (**A**) Comparison between BC−10% and CK. (**B**) Comparison between BC−20% and CK. Blue boxes indicate pathways commonly enriched in both BC−10% vs. CK and BC−20% vs. CK, while red boxes indicate pathways enriched in BC−20% vs. CK but not in BC−10% vs. CK. The box−labeled pathways represent the specific pathways discussed in the main text, rather than the full set of all enriched terms.

To further characterize the biological pathways regulated by bamboo-compost amendment, Kyoto Encyclopedia of Genes and Genomes (KEGG) pathway enrichment analysis was performed (Supplementary Table S6). Among the top 20 significantly enriched pathways, 13 were commonly enriched in both the BC−10% and BC−20% treatments (Figure 9, blue, Supplementary Table S9). These shared pathways included plant–pathogen interaction, MAPK signaling pathway–plant, plant hormone signal transduction, photosynthesis−antenna proteins, sphingolipid metabolism, fatty acid elongation, pentose and glucuronate interconversions, alpha-linolenic acid metabolism, brassinosteroid biosynthesis, arachidonic acid metabolism, phenylpropanoid biosynthesis, glycosphingolipid biosynthesis–ganglio series, and other glycan degradation. Collectively, these results indicate that bamboo-compost amendment was associated with changes in defense responses, lipid metabolism, cell-wall-related metabolic processes, and hormone-associated signal transduction.

In contrast, the BC−20% treatment exhibited unique enrichment of several pathways associated with structural barrier biosynthesis, lipid remodeling, and physiological adaptation (Figure 9, red, Supplementary Table S9). These uniquely enriched pathways included glycerophospholipid metabolism, circadian rhythm–plant, cutin, suberine, and wax biosynthesis, glycosphingolipid biosynthesis–globo and isoglobo series, carotenoid biosynthesis, biosynthesis of unsaturated fatty acids, and glycerolipid metabolism. Notably, the cutin, suberine, and wax biosynthesis pathway contributes to the formation of the hydrophobic leaf cuticle, and its enrichment suggests that a higher proportion of bamboo compost may reinforce structural barriers associated with resistance to insect herbivory. Overall, these findings indicate that the BC−20% treatment was associated with broader and more complex transcriptional reprogramming in tomato plants, particularly involving structural defense-related lipid metabolism and the biosynthesis of protective secondary metabolites.

### 2.10. Differentially Expressed Transcription Factor-Encoding Genes

**Figure 10:**
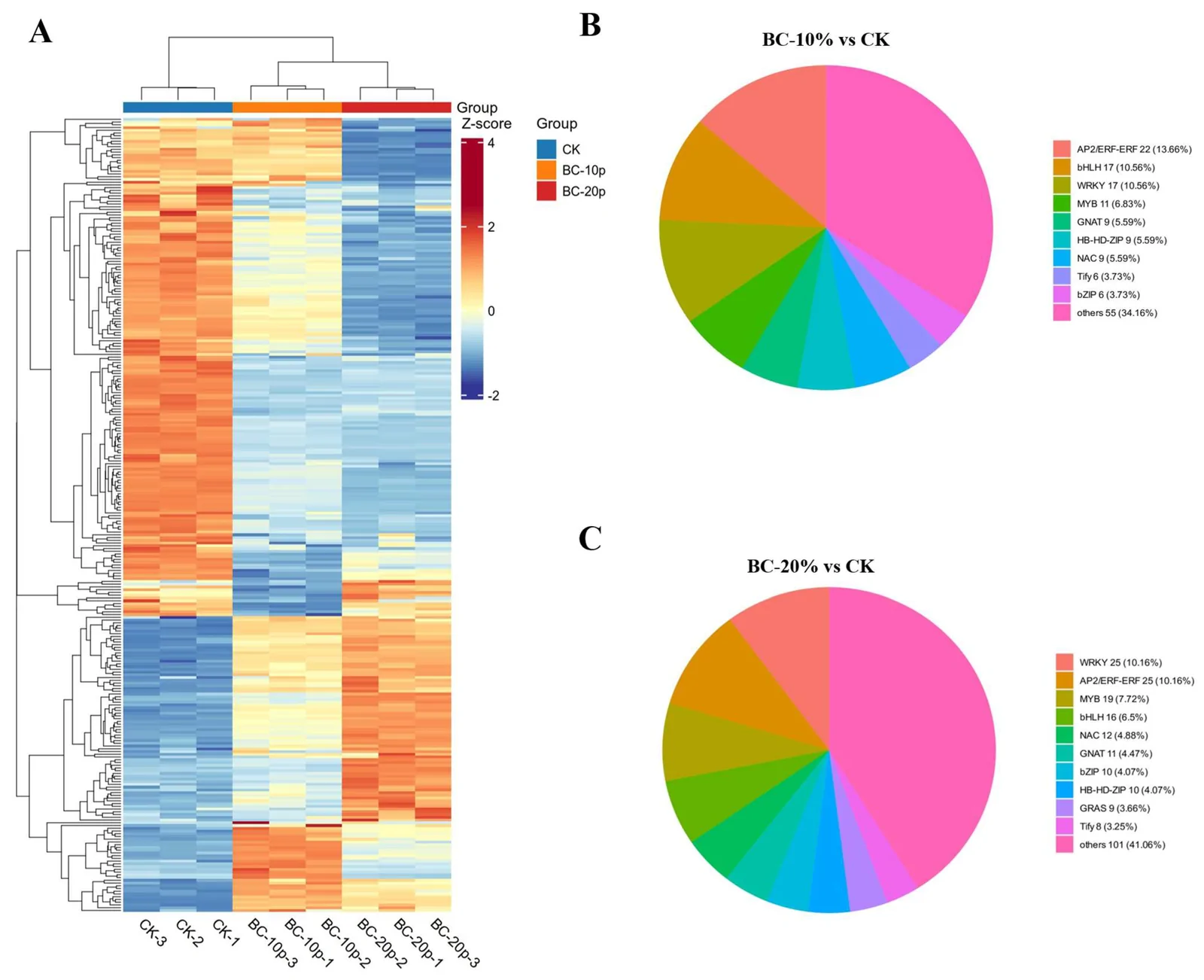
(**A**) Differentially expressed genes encoding transcription factors (TF−DEGs). (**B**) Classification of transcription factor families in the comparison between BC−10% and CK. (**C**) Classification of transcription factor families in the comparison between BC−20% and CK.

Transcription factors (TFs) are key regulators that enable plants to perceive environmental changes and modulate downstream gene expression, playing critical roles in growth, development, phytohormone signaling, secondary metabolism, and defense against biotic and abiotic stresses. To further characterize the effects of bamboo compost on transcriptional regulatory networks in tomato leaves, TF annotation was performed on the differentially expressed genes identified in the BC−10% and BC−20% treatments relative to CK. A total of 290 differentially expressed TF−encoding genes (TF−DEGs) were identified across the two treatment comparisons (Supplementary Table S7a). Heatmap clustering revealed distinct TF−DEG expression patterns among the CK, BC−10%, and BC−20% samples, whereas biological replicates within each treatment clustered closely, indicating that bamboo compost markedly altered the expression profiles of TF−encoding genes in tomato leaves (Figure 10A).

In the BC−10% vs. CK comparison, 161 TF−DEGs were identified, comprising 65 upregulated and 96 downregulated genes (Supplementary Table S7b). These TF−DEGs were predominantly assigned to the AP2/ERF−ERF (22 genes, 13.66%), bHLH (17 genes, 10.56%), WRKY (17 genes, 10.56%), MYB (11 genes, 6.83%), GNAT (9 genes, 5.59%), HB−HD−ZIP (9 genes, 5.59%), NAC (9 genes, 5.59%), TIFY (6 genes, 3.73%), and bZIP (6 genes, 3.73%) families, with the remaining 55 genes (34.16%) belonging to other TF families (Figure 10B). The relatively high representation of the AP2/ERF−*ERF*, *WRKY*, *bHLH*, *MYB*, and *NAC* families suggests that the BC−10% treatment was associated with a substantial transcriptional regulatory response in tomato leaves.

In contrast, the BC−20% treatment was associated with more extensive changes in TF expression. A total of 246 TF-DEGs were identified in the BC−20% vs. CK comparison, including 86 upregulated and 160 downregulated genes (Supplementary Table S7c). These genes were mainly assigned to the WRKY (25 genes, 10.16%), AP2/ERF-ERF (25 genes, 10.16%), MYB (19 genes, 7.72%), bHLH (16 genes, 6.50%), NAC (12 genes, 4.88%), GNAT (11 genes, 4.47%), bZIP (10 genes, 4.07%), HB-HD-ZIP (10 genes, 4.07%), GRAS (9 genes, 3.66%), and TIFY (8 genes, 3.25%) families, with the remaining 101 genes (41.06%) assigned to other TF families (Figure 10C). Compared with BC−10%, the BC−20% treatment yielded a greater number of TF-DEGs and increased representation of the WRKY, AP2/ERF-ERF, MYB, NAC, bZIP, and GRAS families, indicating that the higher bamboo-compost proportion induced more extensive transcriptional reprogramming.

Comparative analysis revealed 117 TF−DEGs shared between the BC−10% and BC−20% treatments, of which 42 were upregulated in both. These commonly upregulated genes included five NAC genes (*Solyc01G003485.ITAG5.0*, *Solyc02G000676.ITAG5.0*, *Solyc02G001198*.*ITAG5.0*, *Solyc02G002858.ITAG5.0*, and *Solyc05G001366.ITAG5.0*), four MYB genes (*Solyc05G002084.ITAG5.0*, *Solyc06G000030.ITAG5.0*, *Solyc06G000031.ITAG5.0*, and *Solyc10G002910.ITAG5.0*), four bHLH genes (*Solyc01G003852.ITAG5.0*, *Solyc06G000187.ITAG5.0*, *Solyc12G000302.ITAG5.0*, and novel.710), three AP2/ERF-ERF genes (*Solyc01G002624.ITAG5.0*, *Solyc03G000249.ITAG5.0*, and *Solyc12G002257.ITAG5.0*), and three WRKY genes (*Solyc02G002411.ITAG5.0*, *Solyc05G001069.ITAG5.0*, and *Solyc12G001884.ITAG5.0*). These commonly upregulated members of the NAC, MYB, bHLH, AP2/ERF-ERF, and WRKY families may constitute an important transcriptional regulatory module involved in bamboo-compost-induced defense responses and metabolic regulation in tomato leaves.

Conversely, 74 TF−DEGs were downregulated in both treatments, primarily belonging to the WRKY (10 genes), AP2/ERF-ERF (8 genes), GNAT (7 genes), bHLH (6 genes), TIFY (6 genes), MYB (5 genes), HB-HD-ZIP (4 genes), and bZIP (3 genes) families. Notably, six TIFY genes (*Solyc03G003507.ITAG5.0*, *Solyc06G002732.ITAG5.0*, *Solyc08G000634.ITAG5.0*, *Solyc08G000635.ITAG5.0*, *Solyc08G000637.ITAG5.0*, and *Solyc12G000335.ITAG5.0*) were consistently downregulated in both treatments, suggesting that bamboo compost may exert a stable regulatory effect on jasmonic acid-related TIFY/JAZ components. In addition, one HB-KNOX gene (*Solyc02G001771.ITAG5.0*) was downregulated in the BC−10% treatment but upregulated in the BC−20% treatment, indicating a potentially strong dose-dependent response to the bamboo-compost proportion.

Treatment-specific analysis identified 44 TF-DEGs unique to BC−10%, including 23 upregulated and 21 downregulated genes, mainly belonging to the AP2/ERF-ERF, bHLH, WRKY, MYB, and NAC families. By comparison, 129 TF-DEGs were unique to BC−20%, comprising 43 upregulated and 86 downregulated genes, predominantly from the AP2/ERF-ERF, WRKY, MYB, GRAS, bHLH, NAC, and bZIP families. Overall, the two bamboo−compost proportions exerted shared but quantitatively distinct effects on TF expression in tomato leaves. The BC−10% treatment primarily induced responses in a subset of core defense-related TFs, whereas BC−20% affected a larger number and broader range of TF families, particularly WRKY, AP2/ERF-ERF, MYB, NAC, bHLH, bZIP, and GRAS. These results suggest that a higher proportion of bamboo compost may further enhance transcriptional regulatory responses associated with phytohormone signaling, secondary metabolism, and insect defense in tomato leaves.

### 2.11. Changes in Genes and Metabolites Associated with MAPK and Phytohormone Signaling

Bamboo compost treatment was associated with differential expression of numerous genes involved in the MAPK signaling pathway (ko04016) and the plant hormone signal transduction pathway (ko04075). Treatment with BC−10% and BC−20% led to the differential expression of 75 and 103 MAPK pathway-related genes and 105 and 158 hormone signaling-related genes, respectively (Supplementary Table S10). Several genes were significantly upregulated in the BC−10% treatment, including those encoding leucine-rich repeat receptor-like serine/threonine-protein kinase FLS2/EFR (e.g., *Solyc06G000833* and *PRAM_24043*), the brassinosteroid insensitive 1−associated receptor kinase BAK1/SERK3 (*Solyc01G000410*), and the transcription factor *MYC2* (*Solyc01G003852*). Furthermore, *Solyc02G001948*, annotated as basic endochitinase B (K20547) and mapped to the MAPK signaling pathway (ko04016), exhibited elevated expression. Metabolomic analysis revealed the differential accumulation of hormone signaling-related metabolites, including indole-3-acetate *and* 3′,5′-cyclic GMP, in the BC−20% treatment. These findings indicate that bamboo compost treatment was associated with changes in defense-related receptor, MAPK, and phytohormone signaling components in tomato leaves.

### 2.12. Changes in Genes and Metabolites Associated with Phenylpropanoid, Flavonoid, and Terpenoid Metabolism

Secondary metabolites are essential for plant defense against insect herbivores. Transcriptomic analysis revealed that numerous genes involved in phenylpropanoid and flavonoid biosynthesis were differentially expressed in response to bamboo compost treatment. In the phenylpropanoid biosynthesis (ko00940) and flavonoid biosynthesis (ko00941) pathways, 21 and 11 differentially expressed genes (DEGs) were identified in the BC−10% treatment, respectively, while 33 and 16 DEGs were detected in the BC−20% treatment. Key biosynthetic genes, including *chalcone synthase* (*Solyc09G002676*), flavanone 3-dioxygenase *(PRAM_26184),* and caffeoyl-CoA O-methyltransferase (*Solyc09G002401*), were significantly upregulated in the BC−10% treatment. Metabolomic analysis further corroborated these transcriptional changes, with the identification of 99 flavonoid and 89 phenolic acid differentially accumulated metabolites (DAMs). The BC−20% treatment yielded a greater number of upregulated flavonoid DAMs (32) than the BC−10% treatment (14); these included flavonol glycosides associated with quercetin and kaempferol that exhibited differential accumulation (Supplementary Table S11).

Transcriptomic analysis also demonstrated that genes participating in terpenoid backbone biosynthesis (ko00900) and downstream monoterpenoid, diterpenoid, and sesquiterpenoid pathways were modulated by bamboo compost (Supplementary Table S11). For instance, geranylgeranyl pyrophosphate synthase 1 (*Solyc11G000507*) was significantly upregulated in the BC−20% treatment, whereas several hydroxymethylglutaryl-CoA reductase (HMGR) genes were downregulated. At the metabolite level, 79 terpenoid DAMs were identified, and the BC−20% treatment was associated with the differential accumulation of 21 monoterpenoids, 17 diterpenoids, and 28 sesquiterpenoids. The coordinated transcriptomic and metabolomic changes were associated with altered phenylpropanoid, flavonoid, and terpenoid metabolism, which may be related to reduced *Tuta absoluta* larval survival.

### 2.13. Changes in Genes and Metabolites Associated with Fatty Acid Metabolism, Cutin and Wax Biosynthesis, and Alkaloid Metabolism

Fatty acid metabolism and the formation of surface barriers serve as important defenses against insect feeding. Within the alpha-linolenic acid metabolism pathway (ko00592), which provides precursors for jasmonic acid biosynthesis, phospholipase A1 genes (*Solyc02G001498* and *Solyc11G002006*) and a lipoxygenase gene (*Solyc12G000514*) were significantly upregulated in the BC−10% treatment. Furthermore, the cutin, suberine, and wax biosynthesis pathway (ko00073) contained 4 and 11 differentially expressed genes (DEGs) in the BC−10% and BC−20% treatments, respectively (Supplementary Table S12). Several 3−ketoacyl-CoA synthase genes, including *Solyc05G000778* and *Solyc02G002197*, were significantly upregulated in both treatments and were annotated to the fatty acid elongation pathway (ko00062). These findings indicate that bamboo compost influenced lipid metabolism and pathways associated with surface-barrier biosynthesis. However, the specific contributions of these 3-ketoacyl-CoA synthases to cutin and wax formation require functional validation.

Alkaloids represent another important class of insect-defense compounds. A total of 95 alkaloid differentially accumulated metabolites (DAMs) were identified, including 31 phenolamines that exhibited differential accumulation in the BC−20% treatment (Supplementary Table S12). Genes associated with tropane, piperidine, and pyridine alkaloid biosynthesis (ko00960) and the biosynthesis of various alkaloids (ko00996), such as *Solyc05G002477* and *Solyc10G000297*, were differentially expressed. These transcriptional changes were accompanied by the differential accumulation of alkaloid metabolites, including L−hyoscyamine.

### 2.14. Integrated Transcriptomic and Metabolomic Analysis

**Figure 11:**
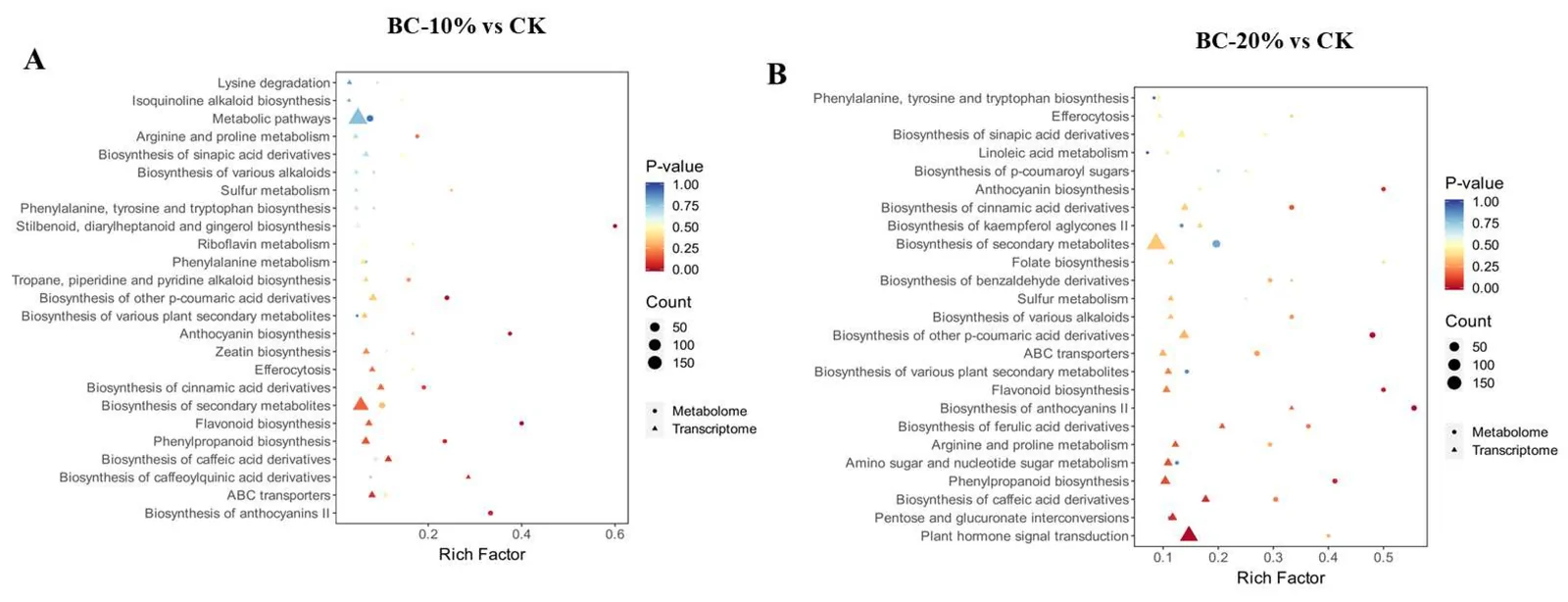
Common KEGG pathway analysis of differentially expressed genes (DEGs) and differentially accumulated metabolites (DAMs) in tomato leaves under bamboo compost treatments. (**A**) BC−10% vs. CK. (**B**) BC−20% vs. CK. Circles represent metabolomic results, whereas triangles represent transcriptomic results. The *x*-axis indicates the enrichment factor, symbol size represents the number of DEGs or DAMs mapped to each pathway, and color indicates the enrichment *p*-value.

To formally integrate the transcriptomic and metabolomic datasets, DEGs and DAMs from the BC−10% vs. CK and BC−20% vs. CK comparisons were mapped to common KEGG pathways (Figure 11; Supplementary Table S13). In BC−10% vs. CK, phenylpropanoid biosynthesis, flavonoid biosynthesis, and secondary-metabolite biosynthesis jointly contained 21, 11, and 123 DEGs, together with 4, 4, and 17 DAMs, respectively. In BC−20% vs. CK, the corresponding pathways included 33, 16, and 193 DEGs and 7, 5, and 33 DAMs; plant hormone signal transduction additionally comprised 158 DEGs and 2 DAMs.

Pearson’s correlation analysis of matched gene-expression and metabolite-abundance profiles identified DEG–DAM associations at |r| > 0.8 and *p* < 0.05. In BC−20% vs. CK, 133 associations were detected in phenylpropanoid biosynthesis and 99 in plant hormone signal transduction (Supplementary Tables S14 and S15). These results indicate coordinated transcriptomic and metabolic responses to bamboo compost.

## 3. Discussion

This study demonstrates that bamboo compost, as a renewable organic amendment for growing substrates, simultaneously influences tomato seedling growth, leaf biochemical metabolism, and resistance to the South American tomato leafminer, *Tuta absoluta*. Replacing peat and valorizing agricultural organic waste have become key strategies for sustainable protected horticulture. However, there is still limited systematic evidence regarding optimal application rates of organic substrate materials and their regulatory effects on plant resistance to insect pests [45,46]. In this study, the BC−10% treatment significantly increased tomato plant height and stem diameter, whereas BC−20% and BC−30% inhibited seedling growth. Chlorophyll content decreased to varying degrees across all bamboo compost treatments. Because physicochemical properties of the treatment substrates were not measured in the present study, the growth reduction observed at higher bamboo-compost proportions cannot be attributed to a specific factor, such as pH, EC, porosity, nutrient availability, or C/N ratio. Previous studies have shown that the physicochemical properties of bamboo-residue compost and compost-based growing media can vary with feedstock, composting conditions, and blending ratio, and can influence their suitability as peat substitutes [47]. Future studies should therefore characterize treatment-specific substrate properties together with plant responses. Previous bamboo-charcoal studies reported enhanced tomato growth and insect resistance together with altered defense-related metabolites [48]. The present study extends this work by evaluating bamboo compost, a microbially decomposed organic amendment, across a 5−30% incorporation gradient in a peat-reduced substrate. Rather than proposing a compost-exclusive defense pathway, our findings define a dose-dependent biological pattern: BC−10% was associated with the most favorable seedling-growth phenotype, whereas BC−20% was associated with lower larval survival and broader transcriptomic and metabolic reprogramming. Although MAPK/phytohormone signaling and phenylpropanoid/flavonoid-related pathways overlapped with those reported for bamboo charcoal, the present results identify a growth−defense association specific to the bamboo-compost dose–response context. Direct bamboo compost–bamboo charcoal comparisons will be required to determine whether their underlying mechanisms are distinct.

Importantly, bamboo compost significantly reduced the survival of *Tuta absoluta* larvae on tomato leaves, consistent with enhanced plant resistance. *Tuta absoluta* is a major invasive pest of tomatoes worldwide. Its concealed feeding behavior, rapid reproduction, and ability to develop insecticide resistance highlight the need for sustainable management strategies that leverage endogenous plant resistance [49]. Previous studies have shown that biochar and other organic amendments can improve plant resistance to pests and diseases by modifying rhizosphere microbial communities, inducing systemic resistance, and altering plant metabolic status [50,51]. A global meta-analysis also demonstrated that biochar application generally reduced plant disease severity and increased plant biomass, although these effects varied with feedstock, application rate, and crop species [52]. Moreover, bamboo-charcoal soil amendment exerted bottom−up effects on tomato–whitefly interactions, reducing insect survival, oviposition, and field population density [53]. In this study, the BC−10%, BC−15%, and BC−20% treatments significantly reduced *Tuta absoluta* survival. This finding suggests that reduced larval survival was not simply associated with improved plant growth, but may be associated with treatment-related changes in defense metabolism and signaling pathways.

The biochemical and metabolomic data further support this interpretation. Bamboo compost significantly altered the concentrations of soluble sugars, soluble proteins, total amino acids, tannins, total phenolics, and cell wall-related components, as well as the activities of phenylalanine ammonia-lyase (PAL) and catalase (CAT), in tomato leaves. PAL catalyzes a key entry step in the phenylpropanoid pathway, and increased PAL activity is generally associated with an enhanced capacity to synthesize defensive compounds such as phenolic acids, flavonoids, lignin, and tannins. PAL activity peaked under BC−5% but declined under BC−10%, whereas flavonoid content increased under both treatments. This difference may reflect the transient nature of enzyme activity relative to the cumulative accumulation of downstream metabolites. Likewise, reduced tannin content may indicate a shift in phenylpropanoid flux toward other defense-related metabolites rather than a general decline in defense capacity. The distinct responses of sugars, proteins, and amino acids further indicate dose-dependent adjustment of primary metabolism associated with defense reprogramming [54,55]. Metabolomic analysis identified 292 and 591 differentially accumulated metabolites (DAMs) in the BC−10% and BC−20% treatments, respectively. These metabolites mainly included amino acids and their derivatives, flavonoids, alkaloids, phenolic acids, terpenoids, lipids, and steroids. Plant secondary metabolites, particularly flavonoids, phenylpropanoids, terpenoids, and alkaloids, can contribute to insect resistance by deterring feeding, disrupting digestive metabolism, and strengthening antioxidant defenses [56,57]. Previous studies of tomato *Tuta absoluta* interactions have similarly shown that leafminer feeding induces marked changes in phenolamine, phenylpropanoid, and flavonoid metabolic pathways [58,59]. Therefore, the accumulation of secondary metabolites associated with bamboo compost treatment may contribute to the reduced larval survival observed in this study.

Transcriptomic analysis provided deeper insights into the molecular processes underlying bamboo-compost-associated resistance. The BC−10% and BC−20% treatments resulted in 1553 and 2591 differentially expressed genes (DEGs), respectively, with 1156 DEGs shared between the two treatments. These findings indicate that bamboo compost induces extensive transcriptional reprogramming in tomato leaves. Gene Ontology (GO) and Kyoto Encyclopedia of Genes and Genomes (KEGG) enrichment analyses revealed that both treatments affected pathways related to defense responses, wound responses, jasmonic acid signaling, MAPK signaling, plant hormone signal transduction, plant−pathogen interactions, phenylpropanoid biosynthesis, alpha-linolenic acid metabolism, and lipid metabolism. These pathways represent central components by which plants perceive external stimuli and trigger defense responses. Notably, MAPK and phytohormone signaling can transduce rhizosphere- or leaf-derived signals into systemic defense responses, while jasmonic acid and its fatty acid precursor pathways are closely linked to resistance against chewing and leaf-mining insects [60,61]. Genes encoding LRR receptor-like serine/threonine−protein kinases, BAK1/SERK, PP2C, MYC2, GH3, SAUR, PYR/PYL, and GID1 were differentially expressed in the MAPK and phytohormone signaling pathways. These findings suggest that changes in receptor kinase, MAPK, and phytohormone-related pathways may be associated with enhanced tomato resistance. Notably, higher bamboo compost treatments resulted in greater numbers of differentially expressed genes (DEGs) and differentially accumulated metabolites (DAMs), along with a larger proportion of downregulated genes. Therefore, BC−20% was associated with broader defense−related and metabolic changes, consistent with a growth−defense trade-off. This pattern may explain why BC−10% was more favorable for seedling growth, whereas BC−20% was associated with lower larval survival.

Coordinated changes in phenylpropanoid, flavonoid, and terpenoid metabolism were among the most prominent defense−related features observed in this study. Bamboo compost was associated with changes in pathways involved in phenylpropanoid, flavonoid, terpenoid backbone, diterpenoid, and sesquiterpenoid/triterpenoid biosynthesis. These effects were accompanied by altered expression of genes encoding chalcone synthase, flavonoid-modifying enzymes, cinnamoyl-CoA reductase-like proteins, geranylgeranyl pyrophosphate synthase, β-amyrin synthase, and cytochrome P450 enzymes. Thus, bamboo compost may be associated with changes in against *Tuta absoluta* by boosting the activity of phenylpropanoid, flavonoid, and terpenoid metabolic networks. Additionally, pathways related to fatty acid metabolism, cutin, suberine, and wax biosynthesis, unsaturated fatty acid biosynthesis, and glycerophospholipid metabolism were significantly enriched in the BC−20% treatment. This finding suggests that a higher proportion of bamboo compost may also impact the epidermal barrier of tomato leaves. The plant cuticle and its waxes not only limit water loss but also serve as a first line of defense against insect attachment, feeding, and oviposition [62]. A recent metabolomic study of insect-resistant wild tomato accessions similarly identified fatty acids and related metabolic pathways as important components of resistance to whiteflies and *Tuta absoluta* [63]. In this study, the differential expression of 3-ketoacyl-CoA *synthase*, lipoxygenase, phospholipase, and genes related to cutin and wax, along with changes in lipid- and wax-related metabolites, suggests that bamboo-compost-associated resistance may involve both the production of defensive chemicals and the reinforcement of structural barriers.

Transcription factor analysis provided further evidence for the involvement of a complex regulatory network in bamboo−compost−associated resistance. Across the BC−10% and BC−20% treatments, 290 differentially expressed transcription factor-encoding genes (TF-DEGs) were identified, mainly from the AP2/ERF-ERF, WRKY, bHLH, MYB, NAC, bZIP, GRAS, and TIFY families. Members of the WRKY, MYB, bHLH, NAC, and AP2/ERF families frequently regulate phytohormone signaling, defense responses, and secondary metabolism [64]. The BC−20% treatment induced more TF−DEGs than BC−10% and caused broader changes in defense-related families, including WRKY, AP2/ERF-ERF, MYB, and NAC. This indicates a stronger transcriptional regulatory response at the higher bamboo compost proportion. Combined with the metabolomic data, these findings indicate that bamboo compost treatment was associated with coordinated changes in signaling, transcriptional regulation, and secondary metabolism that may be related to enhanced tomato resistance. Overall, the results show a dose-dependent association between bamboo compost, tomato seedling growth, and *Tuta absoluta* larval survival. BC−10% was associated with improved seedling growth, whereas BC−20% was associated with broader transcriptomic and metabolic reprogramming and lower larval survival. These associations may involve coordinated changes in MAPK and phytohormone signaling, jasmonic-acid-related lipid metabolism, secondary metabolism, cutin and wax barriers, and defense-related transcription factors. Although the present study identifies dose-dependent growth and defense responses associated with bamboo compost, several limitations should be considered when interpreting the underlying mechanisms. The multi−omics results are correlative, and treatment-specific substrate properties, compost-associated microorganisms, phytohormone dynamics, and the functions of key genes were not directly assessed. Moreover, the detached leaf assay and use of a single tomato cultivar under greenhouse conditions may limit extrapolation to whole-plant and field resistance. Future studies integrating these measurements and functional analyses, together with direct comparisons between bamboo compost and bamboo charcoal, will help define their distinct contributions to tomato growth and insect resistance [65].

## 4. Materials and Methods

### 4.1. Plant Material and Growth Conditions in Bamboo-Compost-Amended Substrates

Tomato cv. “Alias” (*Solanum lycopersicum L*.) was used in this study. Seeds were obtained from the Zhejiang Academy of Agricultural Sciences, China. The experiment was conducted in 2026. After surface sterilization, seeds were sown in square pots (30 cm wide × 37 cm deep) filled with coconut coir and maintained in an insect-free greenhouse at the experimental station of the Zhejiang Academy of Agricultural Sciences. Day/night temperatures were maintained at 24 ± 1 °C/20 ± 1 °C, and relative humidity was 60 ± 5%. No insecticides or herbicides were applied throughout the experiment. Plants in both the treatment and control groups received 2 g per pot of a water-soluble nitrogen–phosphorus–potassium (N–P–K) fertilizer (OMEX, 18−18−18). At the two-leaf stage, seedlings were transplanted into substrates containing different volume-based proportions of bamboo compost. The ratios of bamboo compost, peat, perlite, and vermiculite were 5:65:20:10 (BC−5%), 10:60:20:10 (BC−10%), 15:55:20:10 (BC−15%), 20:50:20:10 (BC−20%), and 30:40:20:10 (BC−30%), respectively. A substrate composed of perlite, vermiculite, and peat at a ratio of 20:10:70 (*v*/*v*/*v*) served as the control (CK). Each treatment included three biological replicates, with five plants per replicate and 15 plants in total. Plants were watered every 2 days. At 20 days after transplantation, plant height was measured from the substrate surface to the shoot apex using a ruler, stem diameter was measured using a digital Vernier caliper (MNT919210, Meinaite, Shanghai, China), and leaf chlorophyll content was estimated using a SPAD chlorophyll meter (SPAD−502Plus, Konica Minolta, **Tokyo,** Japan) [66].

### 4.2. Feeding Bioassay and Survival of Tuta absoluta on Tomato Plants Grown in Bamboo-Compost-Amended Substrates

A laboratory colony of the South American tomato leafminer (*Tuta absoluta*) was originally collected in Ili, Xinjiang, China, in 2018 and continuously maintained in a controlled-environment chamber at 25 ± 1 °C, 60 ± 5% relative humidity, and a 16:8 h (light:dark) photoperiod. Tomato plants exhibiting uniform growth were selected from each treatment, and the third fully expanded true leaf from the apex was excised using scissors. The petiole of each detached leaf was inserted through a perforated cap into a 2−mL microcentrifuge tube containing distilled water, with the base of the petiole kept submerged throughout the experiment to maintain leaf hydration and freshness. Each leaf–tube assembly was placed in a 90-mm-diameter Petri dish and maintained at 25 ± 1 °C. For each treatment, 50 s−instar *Tuta absoluta* larvae were individually reared on separate detached leaves, each constituting an independent experimental unit. Leaves from the corresponding tomato treatments were renewed every 2 days, and larval survival was recorded daily for 10 days.

### 4.3. Determination of Physiological and Biochemical Parameters

Nine physiological and biochemical parameters were evaluated: soluble sugar content, soluble protein content, total amino acid content, PAL activity, CAT activity, tannin content, total phenolic content, hemicellulose content, and total flavonoid content. Soluble sugar content, soluble protein content, and total amino acid content were determined using the anthrone colorimetric method, bicinchoninic acid (BCA) assay, and ninhydrin colorimetric method, respectively [67]. Total flavonoid content was measured using the sodium nitrite−aluminum nitrate colorimetric method [68]. Total phenolic and tannin contents, as well as PAL and CAT activities, were determined using established colorimetric and enzymatic procedures [69]. Hemicellulose content was determined using the 3,5-dinitrosalicylic acid (DNS) colorimetric method [70]. PAL and CAT activities were determined using commercial assay kits (PAL, catalog no. NMW0108; CAT, catalog no. NMK0006; Norminkoda Biotechnology Co., Ltd., Wuhan, China) according to the manufacturer’s instructions. Each biochemical parameter was determined using five independent biological replicates per treatment (n = 5).

### 4.4. Metabolomic Analysis

#### 4.4.1. Sample Preparation and Extraction

Based on the phenotypic screening, the CK, BC−10%, and BC−20% treatments were selected for integrated metabolomic and transcriptomic analyses. BC−10% showed the most favorable seedling-growth phenotype, with the greatest promotion of plant height and stem diameter. In contrast, BC−20% resulted in the lowest *Tuta absoluta* larval survival rate. These two treatments were therefore selected to represent the contrasting growth-promoting and insect-resistance-associated phenotypes induced by bamboo compost. Twenty days after transplantation, fresh third leaves were collected from plants in the CK, BC−10%, and BC−20% treatments, with three biological replicates per treatment. Samples were freeze-dried using a lyophilizer (Scientz-100F, Ningbo Scientz Biotechnology Co., Ltd., Ningbo, China) and ground to a fine powder using a mixer mill (MM 400, Retsch GmbH, Haan, Germany) at 30 Hz for 1.5 min. An aliquot of 30 mg of sample powder was weighed using an electronic balance (MS105DM, Mettler Toledo, Greifensee, Switzerland), and mixed with 1500 μL of pre-cooled (−20 °C) 70% aqueous methanol containing internal standards. The mixture was vortexed for 30 s every 30 min, six times in total. After centrifugation at 12,000 rpm for 3 min, the supernatant was filtered through a 0.22-μm microporous membrane and stored in an injection vial for UPLC-MS/MS analysis.

#### 4.4.2. UPLC Conditions and ESI-QTRAP-MS/MS

The sample extracts were analyzed using an ultra-performance liquid chromatography–electrospray ionization–tandem mass spectrometry (UPLC–ESI–MS/MS) system consisting of an ExionLC™ AD UPLC system (SCIEX, Framingham, MA, USA) coupled to an electrospray ionization triple quadrupole–linear ion trap mass spectrometer (QTRAP; SCIEX, Framingham, MA, USA). The UPLC analytical conditions were as follows: column, Agilent SB-C18 (1.8 μm, 2.1 mm × 100 mm; Agilent Technologies, Santa Clara, CA, USA); mobile phase, solvent A (water with 0.1% formic acid) and solvent B (acetonitrile with 0.1% formic acid). The gradient program was as follows: 0 min, 95% A/5% B; 9 min, linear gradient to 5% A/95% B; held at 5% A/95% B for 1 min; returned to 95% A/5% B over 1.1 min and held for 2.9 min. The flow rate was 0.35 mL/min, the column temperature was maintained at 40 °C, and the injection volume was 2 μL. The effluent was directed to an ESI-triple quadrupole-linear ion trap (QTRAP) mass spectrometer.

The ESI source parameters were as follows: source temperature, 500 °C; ion spray voltage (IS), 5500 V in positive ion mode and −4500 V in negative ion mode; ion source gas I (GSI), gas II (GSII), and curtain gas (CUR) were set at 50, 60, and 25 psi, respectively; collision-activated dissociation (CAD) was set to high. MRM scans were performed with collision gas (nitrogen) set to medium. Declustering potential (DP) and collision energy (CE) for each MRM transition were further optimized. A specific set of MRM transitions was monitored for each period based on the metabolites eluted in that period.

#### 4.4.3. Bioinformatics Analyses of Metabolome Data

Principal component analysis (PCA) was performed using the prcomp function in R (R Foundation for Statistical Computing, Vienna, Austria; www.r-project.org). Pearson’s correlation coefficients between samples were calculated using the cor function in R and visualized as heatmaps. Candidate differentially accumulated metabolites (DAMs) were selected using VIP > 1 and fold-change thresholds of ≥2 or ≤0.5, equivalent to (|log_2_FC| ≥ 1). VIP values were derived from OPLS-DA results generated using the R package MetaboAnalystR and represent the contribution of each metabolite to group discrimination. The data were log_2_-transformed and unit variance-scaled prior to OPLS-DA, and a 200-permutation test was performed to assess potential model overfitting. Univariate *p*-values and FDR values are provided in Supplementary Table S3 for transparency and sensitivity assessment but were not used as the primary candidate-DAM selection criteria. The identified metabolites were annotated using the KEGG Compound database http://www.kegg.jp/kegg/compound/(accessed on 1 June 2026), and the annotated metabolites were then mapped to the KEGG Pathway database http://www.kegg.jp/kegg/pathway.htmlaccessed on 1 June 2026). Pathways to which the differentially accumulated metabolites (DAMs) were mapped were subjected to metabolite set enrichment analysis (MSEA), and significance was determined by hypergeometric test *p*-values.

### 4.5. Transcriptome Sequencing

Fresh third leaves were collected from plants in the CK, BC−10%, and BC−20% treatments, with three biological replicates per treatment, using the same treatment-selection rationale described in Section 4.4.1. Total RNA was extracted using a combination of the cetyltrimethylammonium bromide (CTAB) method (catalog no. A600108-0500, Sangon Biotech Co., Ltd., Shanghai, China) and PBIOZOL reagent (catalog no. BSC55M1, Bioer Technology Co., Ltd.,Hangzhou, China). Genomic DNA contamination was removed using RNase-free DNase I. RNA concentrations were quantified using a Qubit 4.0 fluorometer (Thermo Fisher Scientific, MA, Waltham, USA) with an Equalbit RNA BR Assay Kit (catalog no. EQ212-02, Vazyme Biotech Co., Ltd., Nanjing, China). RNA integrity was evaluated using a Qsep400 high-throughput nucleic acid and protein analyzer (BiOptic Inc., Taiwan, China), and the RNA quality number (RQN) was recorded. All RNA samples used for library preparation met the following quality criteria: OD260/280 = 1.8–2.2, OD260/230 ≥ 2.0, RQN ≥ 7.0, and total RNA amount > 1 μg.

RNA-seq libraries were constructed using the VAHTS Universal V10 RNA-seq Library Prep Kit for Illumina (catalog no. NR616-02, Vazyme Biotech Co., Ltd., Nanjing, China) according to the manufacturer’s instructions. Briefly, poly(A)+ mRNA was enriched using oligo(dT) magnetic beads and fragmented at high temperature in fragmentation buffer. First-strand cDNA was synthesized using the fragmented mRNA as a template, followed by second-strand cDNA synthesis. The resulting double-stranded cDNA was subjected to end repair, A-tailing, and adapter ligation, followed by PCR amplification to generate the final libraries. Library quality and concentration were evaluated before sequencing.

The cDNA libraries were sequenced on an Illumina NovaSeq X platform (Illumina, San Diego, CA, USA) to generate paired-end reads. Raw sequencing reads were processed using fastp version 0.23.2 to remove adapter sequences and low-quality reads. The resulting clean reads were aligned to the *Solanum lycopersicum* ITAG5.0 reference genome using HISAT2 version 2.2.1 with default parameters. The reference genome sequence was obtained from Phytozome v14 (https://phytozome-next.jgi.doe.gov/info/Slycopersicum_ITAG5_0; accessed on 12 May 2026), a plant genomics database developed by the Joint Genome Institute (JGI), USA.

RT-qPCR analysis was performed for the 10 candidate genes using the PrimeScript™.

RT Master Mix (Perfect Real Time) cDNA synthesis kit (Takara, Dalian, China) and TBGreen™ Premix Ex Taq™ II (Tli RNaseH Plus) (Takara, Dalian, China), with GAPDH serving as the reference gene. Relative expression levels were calculated using the 2^−ΔΔCt^ method. Primer sequences are provided in Supplementary Table S16. Three biological replicates and two technical replicates were analyzed for each treatment.

### 4.6. Differentially Expressed Genes and Functional Enrichment Analysis

Transcript assembly was performed using StringTie version 2.1.6. The assembled transcripts were compared with the reference genome annotation using GffCompare to identify novel transcripts. Gene-level raw read counts were obtained using featureCounts. Differential expression analysis was performed using DESeq2 version 1.22.1. Genes with an absolute log2 fold change > 1 and a false discovery rate (FDR) < 0.05 were considered differentially expressed genes (DEGs).

To investigate the potential biological functions of the DEGs, functional annotation was performed using DIAMOND searches against the Kyoto Encyclopedia of Genes and Genomes (KEGG) and Gene Ontology (GO) databases. KEGG pathway and GO term enrichment analyses were subsequently conducted. KEGG pathways and GO terms with an FDR < 0.05 were considered significantly enriched.

#### Integrated Transcriptomic and Metabolomic Analysis

Matched transcriptomic and metabolomic datasets from the same nine samples were used for integrated analysis. DEGs and DAMs from the BC−10% vs. CK and BC−20% vs. CK comparisons were assigned to KEGG pathways, and pathways containing both DEGs and DAMs were identified as common pathways.

Pearson’s correlation coefficients between gene-expression and metabolite-abundance profiles were calculated across the matched samples using the cor function in R. Gene–metabolite pairs with (|r| > 0.8) and *p* < 0.05 were retained for pathway-specific correlation analysis.

### 4.7. Statistical Analysis

Experimental data were initially organized using Microsoft Excel 2019 (Microsoft Corp., Redmond, WA, USA). Statistical analyses were performed using IBM SPSS Statistics version 27.0 (IBM Corp., Armonk, NY, USA). Data that met the assumptions of normality and homogeneity of variance, including day-10 larval survival rates, were analyzed using one-way analysis of variance (ANOVA), followed by Tukey’s honestly significant difference (HSD) test for multiple comparisons. Larval survival curves were compared using the log-rank (Mantel–Cox) test in GraphPad Prism version 10.0 (GraphPad Software, Boston, MA, USA). Differences were considered statistically significant at *p* < 0.05.

## 5. Conclusions

In conclusion, bamboo compost was associated with dose-dependent differences in tomato seedling growth and *Tuta absoluta* larval survival. BC−10% was associated with improved seedling growth, whereas BC−20% was associated with broader defense-related transcriptional and metabolic reprogramming, consistent with a potential growth–defense trade-off. Integrated transcriptomic and metabolomic analyses revealed coordinated changes in MAPK and phytohormone signaling, alpha-linolenic acid metabolism, phenylpropanoid/flavonoid biosynthesis, and cutin and wax-related pathways. These findings provide a pathway-level framework for interpreting bamboo-compost-associated resistance, although the specific regulatory roles of these pathways require further validation. Under the present greenhouse conditions, BC−10% appears to be a promising proportion for peat-reduced tomato substrates. Future studies should integrate functional validation of candidate genes, phytohormone quantification, and rhizosphere microbiome analysis with evaluations across tomato cultivars, bamboo compost sources, and whole-plant and field conditions.

## Figures and Tables

**Table 1:** Summary of Illumina RNA-sequencing data.

Samples	Clean Reads (Reads)	Clean Base(G)	GC Content (%)	Q30 (%)	Mapping Reads (%)
CK-1	47,268,852	7.14	42.93	96.45	99.24
CK-2	56,943,598	8.6	42.83	96.42	99.27
CK-3	51,392,158	7.76	42.78	96.38	99.21
BC−10%-1	53,907,356	6.99	42.63	96.44	99.11
BC−10%-2	46,264,384	7.75	42.69	96.32	99.11
BC−10%-3	51,335,304	7.38	42.64	96.36	99.16
BC−20%-1	48,894,958	7.98	42.84	96.38	99.24
BC−20%-2	52,850,814	8.78	42.78	96.31	99.15
BC−20%-3	58,162,916	6.99	42.89	96.4	99.28

## Data Availability

The RNA-seq data generated in this study have been deposited in the NCBI Sequence Read Archive (SRA) under BioProject accession number PRJNA1498230 https://www.ncbi.nlm.nih.gov/sra/PRJNA1498230 (accessed on 21 July 2026).

## Supplementary Materials

The following supporting information can be downloaded at: https://www.mdpi.com/article/10.3390/ijms27188209/s1.
